# Changes in movement patterns in relation to sun conditions and spatial scales in wild western gorillas

**DOI:** 10.1007/s10071-024-01871-9

**Published:** 2024-04-29

**Authors:** B. Robira, S. Benhamou, E. Obeki Bayanga, T. Breuer, S. Masi

**Affiliations:** 1 Centre d’Écologie Fonctionnelle et Évolutive, Université de Montpellier & CNRS, Montpellier, France; 2 Eco-Anthropologie, Centre National de la Recherche Scientifique/Muséum National d’Histoire Naturelle, Université Paris Diderot, Sorbonne Paris Cité, Musée de L’Homme, Paris, France; 3 Associated to Cogitamus Lab,, https://www.cogitamus.fr; 4 Congo Program, Mondika Research Center, Nouabalé-Ndoki National Park, Wildlife Conservation Society, Brazzaville, Republic of the Congo; 5 Wildlife Conservation Society, Global Conservation Program, New-York, USA; 6 World Wide Fund for Nature, Berlin, Germany

**Keywords:** *Gorilla gorilla gorilla*, Landmarks, Place recognition, Planning abilities, Spatial memory, Sun compass

## Abstract

For most primates living in tropical forests, food resources occur in patchworks of different habitats that vary seasonally in quality and quantity. Efficient navigation (i.e., spatial memory-based orientation) towards profitable food patches should enhance their foraging success. The mechanisms underpinning primate navigating ability remain nonetheless mostly unknown. Using GPS long-term tracking (596 days) of one group of wild western lowland gorillas (*Gorilla gorilla gorilla)*, we investigated their ability to navigate at long distances, and tested for how the sun was used to navigate at any scale by improving landmark visibility and/or by acting as a compass. Long episodic movements ending at a distant swamp, a unique place in the home range where gorillas could find mineral-rich aquatic plants, were straighter and faster than their everyday foraging movements relying on spatial memory. This suggests intentional targeting of the swamp based on long-distance navigation skills, which can thus be efficient over a couple of kilometres. Interestingly, for both long-distance movements towards the swamp and everyday foraging movements, gorillas moved straighter under sunlight conditions even under a dense vegetation cover. By contrast, movement straightness was not markedly different when the sun elevation was low (the sun azimuth then being potentially usable as a compass) or high (so providing no directional information) and the sky was clear or overcast. This suggests that gorillas navigate their home range by relying on visual place recognition but do not use the sun azimuth as a compass. Like humans, who rely heavily on vision to navigate, gorillas should benefit from better lighting to help them identify landmarks as they move through shady forests. This study uncovers a neglected aspect of primate navigation. Spatial memory and vision might have played an important role in the evolutionary success of diurnal primate lineages.

**Supplementary Information:**

The online version contains supplementary material available at 10.1007/s10071-024-01871-9.

## Introduction

Tropical forests are characterised by a patchwork of habitats (van Schaik and Brockman 2009). Habitats differ by providing food differing in quantity and quality, and whose availability varies seasonally, resulting in time-dependent habitat preferences for animal species (e.g., Chapman et al. 2003; Pennec et al. 2020). As the habitats may be highly scattered, this may compel animals to occasionally travel several kilometres to reach a particular habitat type and balance their nutrient intakes. This is for example the case in tropical forests for large, inundated areas such as swamps and clearings (aka *bais*), which may attract many large mammals because they offer diverse minerals in large quantities (e.g., Gessner et al. 2014; Magliocca and Gautier-Hion 2004).

When food occurs in dispersed and ephemeral patches outside the forager's perceptual range, a spatiotemporal memory is a prerequisite to forage efficiently (Boyer and Walsh 2010; Grove 2013; Bracis et al. 2015; Robira et al. 2021). Foraging efficiently implies minimising the cost of movements (e.g., moving straight) to accurately target high-quality food while maximising the nutritional benefits of this food. Numerous animal species are expected to rely on a memory of food patch locations and phenology to plan their foraging movements, but what is the actual information used is mainly unknown (Trapanese et al. 2019; Fagan et al. 2013). In addition, especially in primates, movements have mostly been scrutinised for short distances (of some hundreds of metres, for movements from one food tree to another nearby food tree; Trapanese et al. 2019), while animals’ ability to navigate (i.e., to rely on some form of spatial memory to efficiently reach a target beyond perceptual range) at long distances remains poorly investigated. Focusing on the long-distance travels between habitat patches should increase our understanding of the navigation mechanisms across scales.

Independent of the movement scale, the way a mammal can efficiently navigate its home range remains poorly understood. As the home range is a 'familiar area', navigation within it should be supported by mechanisms that are quite different from those at work in animals navigating large unfamiliar areas, as for the long-distance travels of migratory birds during their first migratory journey (Thorup et al. 2007). Depending on the species, animals can rely on three main types of navigation mechanisms (review in Benhamou 2010 and Durieux and Liedvogel 2021), two of which are very useful mainly for navigating unfamiliar areas: using long-distance environmental gradients (Wallraff 2001), and keeping track of the location of the starting point by integrating its own movement (path integration, Mittelstaedt and Mittelstaedt 1980), usually in combination with a compass to keep the process accurate. The third can be operational only for navigating a familiar area: relying on olfactory, auditory, or visual cues that have been integrated into a spatial learning process to become a beacon (a conspicuous object closely associated with a goal, or an intermediate location, along a path leading to a goal) or a landmark (a conspicuous object used to form a reference frame in which the goal location can be addressed and memorised; mosaic map hypothesis, initially proposed by Wallraff 1974).

To navigate their home range, most mammals can thus be expected to rely in some ways on familiar cues, which must be accurately recognised (Tsoar et al. 2011; Warren 2019). In particular, visual landmarks and beacons should be of key importance for visual diurnal foragers such as primates (Dominy et al. 2001; Dominy and Lucas 2001). However, in dense and murky tropical forests with closed canopy, the visual recognition of a place based on local visual cues may largely depend on the lighting conditions, i.e., on whether the sky is clear or overcast.

The lighting level may also affect individuals using a sun compass to get access to a reference direction. A compass is generally favoured when navigating unfamiliar environments, but it can also help organise spatial knowledge about familiar environments when landmark cueing is rendered difficult. In a rainforest environment, where visual panoramas may often be similar and thus difficult to distinguish, young, inexperienced forest human hunter-gatherers inhabiting the dense Congo basin forest use a sun compass, predominantly to navigate outside their familiar range (Jang et al. 2019a, b). The sun azimuth (i.e., the direction of the sun in the horizontal plane) indeed efficiently provides a reference direction and is used by birds (Schmidt-Koenig 1990; Alerstam et al. 2001) and hymenopterans (first demonstration by Santschi 1911; see also Müller & Wehner 2007). Whether diurnal primates are also able to use the sun azimuth to obtain a reference direction has remained completely unexplored.

In this study, we aimed to provide further insights into the navigation ability of primates, using western lowland gorillas (*Gorilla gorilla gorilla*) as a case study, henceforth called western gorillas. Interestingly, the daily movements of this socially cohesive great ape do not include only short-distance travels to nearby food patches (Salmi et al 2020), but also longer journeys towards distant and special forest habitats (i.e., swamp and clearings) to feed on mineral-rich aquatic plants (Nishihara 1995; Magliocca and Gautier-Hion 2002; Metsio et al. 2014; Yamagiwa et al. 2018). These plants are crucial for balancing mineral intakes across seasons in this species (e.g., Masi et al. 2015). A swamp, giving access to unique feeding resources and located at the far periphery of the home range of the study group, provided us with an experimental-like opportunity to address the question of whether western gorillas are also able to navigate efficiently over longer distances. Specifically, we focused on the straightness, duration, and timing of these long-distance movements to test whether they were the simple result of a drift followed by opportunistic feeding, or rather they were intentional and memory-driven.

Western gorillas are known to use memory at short distances to maximise foraging efficiency on high-quality, clustered, and ephemeral resources (Salmi et al. 2020; Robira et al. 2023a). How the target choice is made (i.e., how spatial, temporal, and attribute knowledge is weighted against each other to decide where to go) has recently been mechanistically investigated (Robira et al. 2023b). However, how gorillas manage to go where they want is currently unknown. To obtain further insights into the cognitive processes underlying western gorillas' navigation, we investigated whether the sun may help them better recognize visual cues (landmarks and beacons) and/or as a reference direction, both during short- and long-distance travel. If the sun was used as a navigation tool by an animal, an overcast sky or dense vegetation would result in less efficient navigation because it makes it more difficult to recognise the place (based on beacons and/or landmarks) and/or should induce the inability to use the sun azimuth as a compass. These two putative roles of the sun (cue recognition and sun compass) can be disentangled by investigating to which extent navigation efficiency depends on the sun's elevation because, even in good light conditions, the sun azimuth becomes hard to assess when the sun is high (i.e., at times close to noon; Müller and Wehner 2007). We thus focused on the straightness of movement bouts directed towards feeding patches (a proxy of navigation efficiency) in different contexts of sun elevation and weather conditions to test for the use of a sun compass and/or landmark recognition by western gorillas when they navigate their home range.

## Methods

### Study group and area

The study group ranged close to Mondika Research Station (2°22’N, 16°16’E), in the Djeke triangle, in the western part of the Nouabalé-Ndoki National Park, in the Congo basin. It consisted of 1 silverback, 2 blackbacks, 5 adult females, 2–3 sub-adult males, 1–2 sub-adult females, 1–4 juveniles, and 4–5 infants (following Breuer et al. 2009’s age/sex classes). We tracked the group from dawn to dusk. As gorilla groups are cohesive, we could assess the behaviour of all individuals every day. The study group was fully habituated to human presence for a long time, allowing us to follow the group in very close proximity (ca. 10 m) and thus accurately record their behaviour and the group location without disturbing their daily activities. This group generally travels around 1 km a day, to cover a 7 km^2^ home range (Robira et al. 2022). This home range partially overlaps with neighbouring groups (for example ca. 5% with one monitored neighbouring group, Robira et al. 2022).

**Fig. 1:**
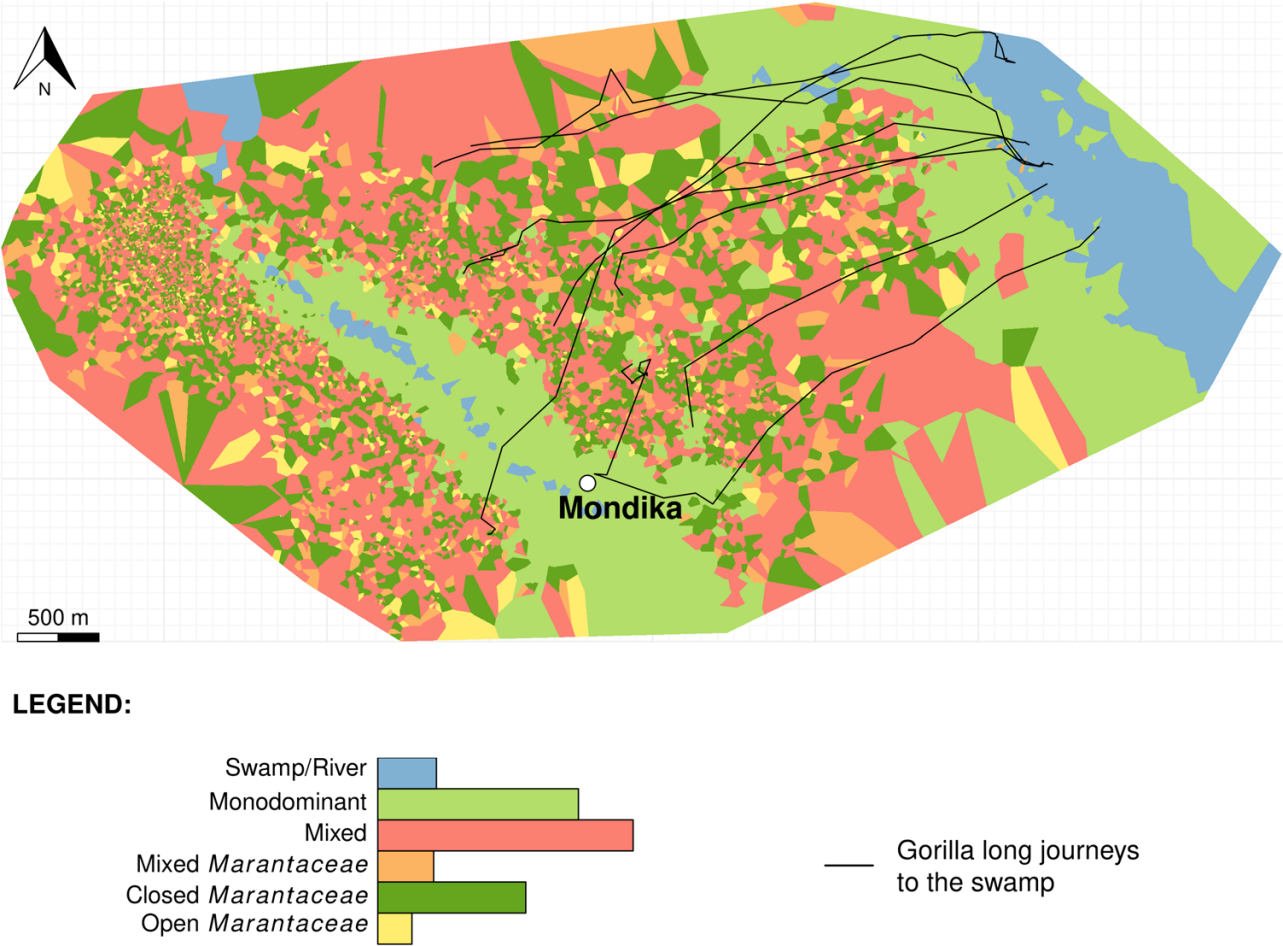
Vegetation distribution in the study area and paths of the study group towards the distant swamp located in the northeastern part of the home range. The vegetation was mapped based on Voronoi (or Dirichlet) tessellation built up from the gorilla GPS locations and the food species eaten (using the ‘voronoi_polygon’ function of the *ggvoronoi* package; Garrett et al. 2022). Contiguous polygons of the same vegetation type were merged. Bar lengths of the histogram are proportional to the area covered by the vegetation. The vegetation includes a monodominant *Gilbertiodendron dewevrei* vegetation, generally found along watercourses or occasionally in large inland stands, mixed-species vegetation (hereafter mixed vegetation), and dense understorey secondary vegetation patches, mixed primary vegetation, mixed *Marantaceae* vegetation, open (i.e., only herbaceous) *Marantaceae* vegetation, closed *Marantaceae* vegetation (with lianas), and swamp/riparian vegetation, hosting aquatic grass. The map was truncated by the Minimum Convex Polygon (MCP) based on all recorded locations, using the ‘mcp’ function of the *adeHabitatHR* package (Calenge 2006), and thus reflects the study group main range. Black tracks represent the long-distance movements reaching the swamp

The area is characterised by the rainfall pattern of a moist tropical forest with a long rainy season from March to November (Supplementary Figure S1). It harbours a patchwork of different vegetation types (Fig. 1). A medium-sized river runs through the centre of the study group’s home range, where it gives birth to a non-swampy riparian area. In addition, a large swamp is located northeast of the group’s home range (Fig. 1). Swamps are an important source of mineral-rich plants for western gorillas (Nishihara 1995; Metsio et al. 2014; Yamagiwa et al. 2018; Magliocca and Gautier-Hion 2002; Doran-Sheehy et al. 2004).

### Locational and behavioural data

Between October 2012 and January 2015 (596 days), the behaviour of all visible individuals of the study group was recorded at 20-min interval scan sampling (Altmann 1974), in synchrony with the GPS location of the group (determined using a handheld Garmin GPS recorder, with an accuracy of 15-18 m), the vegetation openness (canopy and understorey separately; between 0 = very closed to 3 = very open) and type, and the weather conditions (0: mainly blue sky; 1: mainly overcast). These various scores, assessed directly in the field, provide a reliable proxy for the sun illumination as perceived from the ground at the current time. Behaviours were defined as feeding (the food species and type being systematically specified: fruits, young leaves, mature leaves, stems/piths, roots, insects, other), resting (including self-grooming), moving, socialising (e.g., collective play, vocalising) or others (solitary play, vigilance, etc.) (see Masi, et al. 2009 for more details on behavioural classification). We considered that a feeding event occurred when at least three (sub)adults were visible in a given behavioural group scan, and at least half of them were feeding on the same food species (to exclude opportunistic snacking, Salmi et al. 2020). To remove pseudo-movements due to GPS noise when gorillas did not move, we considered the successive GPS locations that were within 30 m of each other as a single location.

**Fig. 2:**
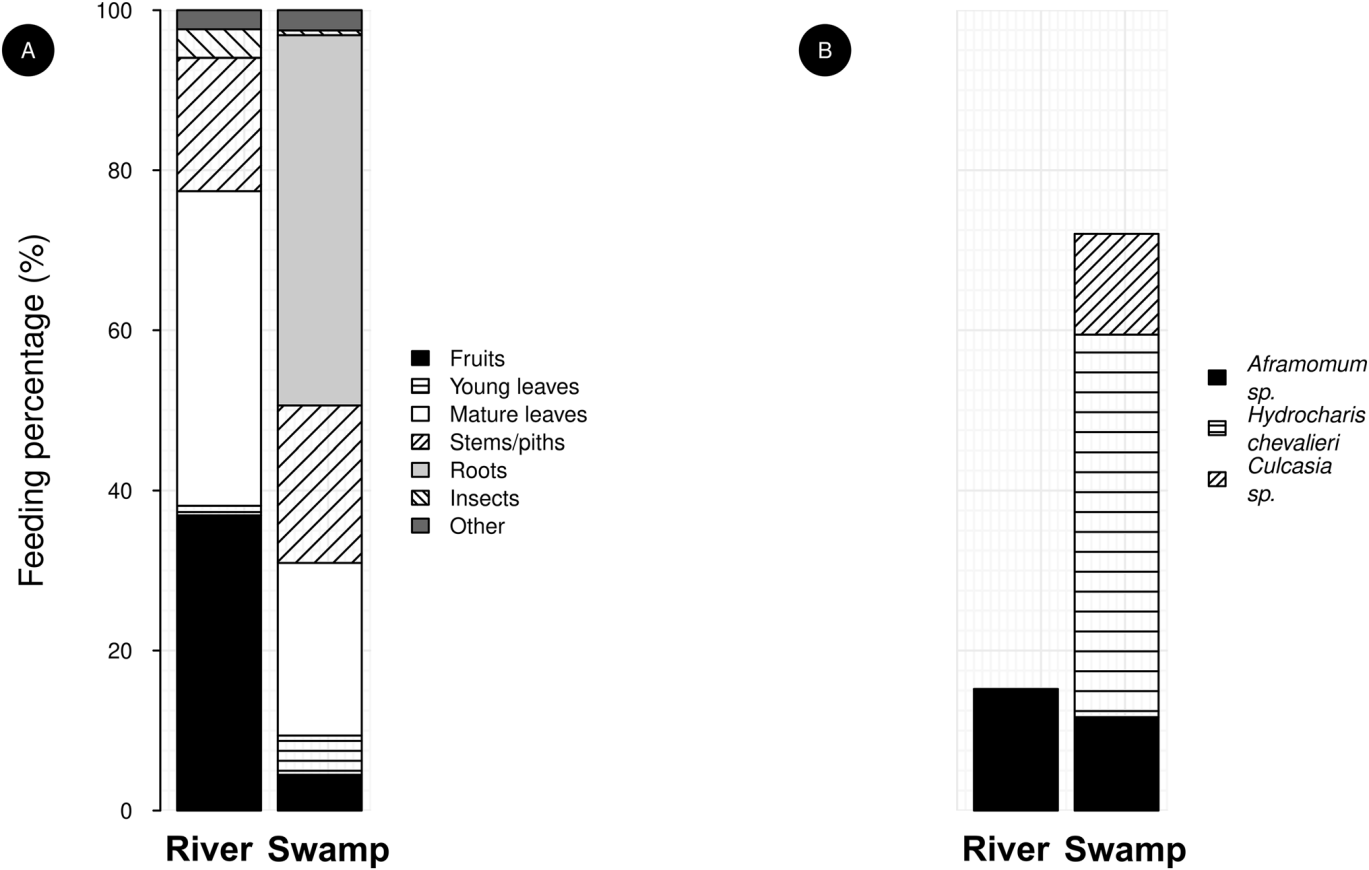
Feeding percentages at the swamp and the river **A** Feeding percentages for each food type as a function of the aquatic habitat **B** Feeding percentages for food species that were mainly eaten in the swamp. Feeding percentage corresponds to the percentage of feeding observations of the food of interest, given all individuals and all feeding observations. Sample (individual-scan) per habitat: River = 79, Swamp = 651

To show that the swamp represents a unique foodspot for the study group, hence worth memorising, we compared food consumption within the swamp and the different habitats, in particular at a river which is located at the heart of their home range (Fig. 1) and represents the only other aquatic habitat, assuming that food consumption would evidence difference in food availability. In each habitat, we calculated the feeding percentage for a given species and/or food type as the proportion of feeding observations on the food of interest given all recorded feeding scans for all individuals. To maximise data independency, we considered data of the same hour as a unique observation. We compared feeding rates with the Horn-Morisita index (adapted for proportions) using the ‘horn_morisita’ function of the *abdiv* package (Bittinger 2020), which accounts for a resemblance in both the rate of consumption and the diversity of the food types. Values close to zero (lower bound) indicate weak similarity, while values close to one (upper bound) indicate high similarity. For this computation, we removed all food types that were not associated with plants (e.g., insects, Fig. 2) and counted each observation (individual-scan) for each species-food type.

### Daily path straightness

In primate studies, the efficiency of navigation is often assessed by two proxies, the path straightness (i.e., the ratio of the beeline distance over the travelled path length) and the movement speed (Janmaat et al. 2021). However, in western gorillas, numerous confounding factors (feeding and social events, resting, reluctance to move in rainy conditions for reasons that are not necessarily related to navigation abilities) may affect the speed. We therefore focused only on the path's straightness to assess navigational efficiency, the speed being considered only as a proxy of the motivation level to quickly reach the target. Following Girard et al. (2004), we looked at the backward evolution of the straightness of the movements that the gorillas performed on a given day. The rationale behind this approach is as follows. When an animal reaches a distant goal, the movement directed towards a given target is expected to be relatively straight, whereas movements performed before this orientation phase starts are more tortuous. Consequently, looking at the backward evolution of the straightness index is an easy means to determine when/where the orientation phase starts: with this approach, intentional movements are evidenced by a straightness, measured backward from the target, that remains very high until reaching the point where the animal decides to move to the target. For the days the gorillas reached the swamp, the backward evolution was computed with respect to the entry point into the swamp. It appeared that gorillas moved straight toward the swamp from locations farther than 1500 m away. We thus considered this as the greatest possible distance for which gorillas showed straight movements toward a given target. Furthermore, 1500 m is the average home range radius observed in western gorillas (including the study group, see Robira et al. 2022). We thus focused specifically on the 8 (out of 41) long-distance (> 1500 m) movements reaching the swamp (and we discarded the gorillas’ shorter movements towards the swamp; see Supplementary Figure S2). We contrasted these movements with the movements performed on all other days, for which the backward evolution was computed with respect to the farthest location reached on that day.

### Segmentation of the daily movements in short-distance movement bouts

Compasses and place recognition may be useful in both short and long-distance navigation, but (1) light conditions can rapidly change along a daily movement, depending on the weather and the local vegetation cover, and (2) the sun elevation is high at midday but far lower during the early morning and late afternoon. For these reasons, we segmented daily movements, irrespective of whether they corresponded to short- or long-distance navigation, in a series of short-term movement bouts to investigate how the sun may affect their straightness. Note that at this short scale, movement bouts are expected to correspond to directed movements in any case, so that this makes sense to estimate their straightness as the ratio beeline distance/path length travelled (Benhamou 2004).

For the days when the swamp was not visited, we defined a movement bout as the movement performed between two successive feeding sites, which should have been deliberately targeted by gorillas based on their spatial memory (Salmi et al. 2020; Robira et al. 2023a). However, these bouts were considered in the analysis only if (1) the starting and ending feeding sites were located at > 50 m away from each other, to avoid considering sensory-driven movement bouts, and (2) there was at least one further GPS location in-between, as the straightness of a bout defined by only two locations is meaningless.

Long-distance movements ending at the swamp could not be segmented into movement bouts in the same way because gorillas rarely stopped to eat on the way. As such movements were clearly directed overall (see Results), we considered any series of three consecutive GPS locations as a movement bout, provided the first and last locations were located > 50 m away from each other. When necessary, additional GPS locations were added to the series of locations until this condition was fulfilled.

### Effect of the lighting level and putative use of the sun azimuth as a compass

All analyses were carried out with the *R* software (v.4.1.2, R Core Team 2020). We tested how the straightness of directed movement bouts may depend on the weather conditions and vegetation openness (canopy and understorey), both acting as proxies for the habitat visibility and thus testing for landmark-based navigation, and the cosine of the sun elevation (a proxy for how easy it is to determine the sun azimuth), to investigate whether gorillas use the sun azimuth as a compass, using a beta regression (Model “Sun visibility” in Supplementary Material, ‘betareg’ function of the *betareg* package, Cribari-Neto and Zeileis 2010; Grün, et al. 2012, with a logit-link function and a fixed dispersion parameter). The sun elevation for each movement bout was determined with the “getSunlightPosition” function of the *suncalc* package (Thieurmel and Elmarhraoui 2022) based on the mid-time of each bout.

Initially, we considered the pairwise interactions between weather (binary variable indicating whether the sky is clear or overcast) and the canopy and understorey openness, because the effect of canopy or understorey openness on the overall visibility may be stronger when the sky is not overcast. We also included an interaction between the weather and the cosine of sun elevation. The use of a sun compass would be highlighted by a significant positive effect of the cosine of sun elevation when the sky was blue (i.e., clear), but no significant effect when the sky was overcast, resulting in a significant interaction between the two factors. We also controlled for the context (swamp *vs* non-swamp days) and the distance travelled during the movement bout (taking the logarithm, and scaling it to a mean of 0 and a standard deviation of 1), since the quality of the measure of straightness may vary with the distance travelled (the GPS locations are sampled only each 20 min).

To ease the final model interpretation, we removed non-significant interactions (but kept the singular variables) and mentioned their respective test in the Results section. This led to the following model formulation

$$f\left(s{\prime}\right)={\beta }_{0}+{\beta }_{1}{I}_{w}+{\beta }_{2}{Z}_{u}+{\beta }_{3}{Z}_{c}+{\beta }_{4}cos\left(e\right)+{\beta }_{5}{Z}_{d}+{\beta }_{6}{I}_{cx}+\epsilon$$

where $s{\prime}=\frac{\left(s\left({N}_{bouts}-1\right)+0.5\right)}{{N}_{bouts}}$, is the adjusted straightness to fit the requirement for a beta regression, with *s* corresponding to the 'standard' straightness and *N*_*bouts*_ to the number of movement bouts considered in the analysis, while *f* is the transformation function required to model a beta regression, ${I}_{w}$ is a binary weather variable (0 for blue sky, 1 for overcast sky), ${Z}_{u}$ and ${Z}_{c}$ are the degrees of openness of the undercover and canopy, respectively (which were z-transformed to a mean of 0 and a standard deviation of 1, for easier interpretability; Schielzeth 2010), cos(*e*) the cosine of the sun’s elevation, ${Z}_{d}$ the logarithm of the distance travelled (also z-transformed), and ${I}_{cx}$ is a context indicator function specifying whether the movement occurred on a “swamp day” or a “non-swamp day”. The $\beta$ values are the regression coefficients to be estimated, and $\epsilon$ is the random error term. After fitting, we verified the necessary statistical assumptions for such modelling (e.g., absence of collinearity, homogeneous distribution of residuals, etc.), the fit quality, and the model stability (i.e., outlier tests). We observed no major issue (see Supplementary Material).

## Results

### The swamp is a source of unique food

Gorillas fed on different food types in the swamp and the riparian area (Fig. 2A; Horn-Morisita similarity index: 0.15). Stems/piths and roots were the main eaten food type in the swamp while gorillas mostly ate fruits and leaves in the riparian area. The two most consumed plants at the swamp (*Hydrocharis chevalieri*, for the roots, and *Culcasia* sp., for the leaves, representing 47.77% and 12.6% of the consumption rate, respectively) were not consumed at the river. The third most consumed plant at the swamp (*Aframomum* sp.) was however eaten at similar rate in both aquatic habitats (Fig. 2B).

### Long-distance movements to the swamp

To go to the swamp, gorillas travelled distances at least three times the daily distance they usually travel (see Supplementary Material “Longer daily travelled distances when the swamp is visited”). Visits to the swamp were not homogeneously distributed during the year (see Supplementary Material “Timing of visits to the swamp area”), with one peak occurring during high frugivory. Nineteen visits (out of 41) were followed by another one in the next three days, with gorillas remaining within 1500 m from the swamp in 84% of the cases. Eight of the total visits started with the group being initially at more than 1500 m from the swamp ([min, max] = [1644, 2461]).

**Fig. 3:**
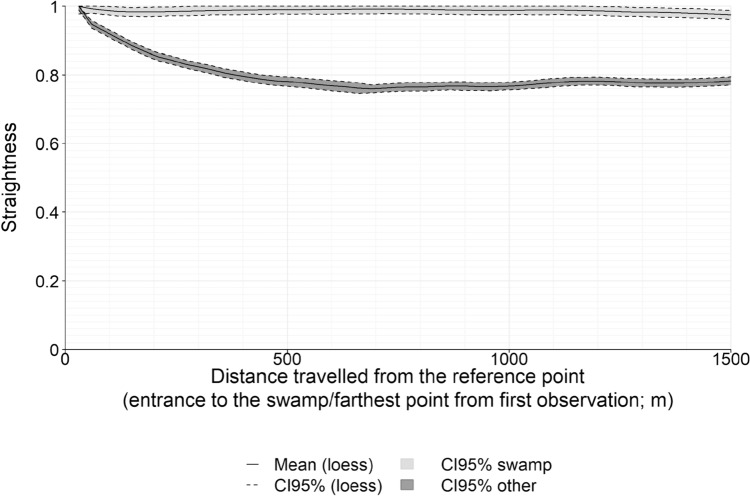
Backward evolution of the straightness as a function of the distance travelled from either the entrance point to the swamp (black lines, light grey background) or the farthest point from the first observation of the day (black lines, dark grey background)**.** In both cases, the plain line corresponds to a local polynomial regression curve (loess; estimated every 30 m with a window span fixed to 10%, ‘loess’ function of the *stats* package, set at default parameters), and the width of the background area indicates the 95% confidence interval

The backward evolution of the straightness of the long-distance movements (> 1500 m) movements ending at the swamp was linear with values very close to 1, indicating a strongly directed movement (Fig. 3). This was markedly different from what was observed for long-distance movements that did not reach the swamp, for which the straightness rapidly decreased as a function of the backward distance to the endpoint (Fig. 3). This means that for everyday movements the ending point was targeted only when the group was close to it, whereas it could be targeted even at more than 1500 m when the ending point corresponded to a swamp entry. During these long-distance movements to the swamp, they ate and rested about 70% less than during movements not ending at the swamp (see Supplementary Material “Reduced feeding/resting opportunities when *en route* to the swamp”). This highlights the high motivational level of the gorillas to quickly reach the swamp once they have decided to go there.

### Influence of the sun

**Fig. 4:**
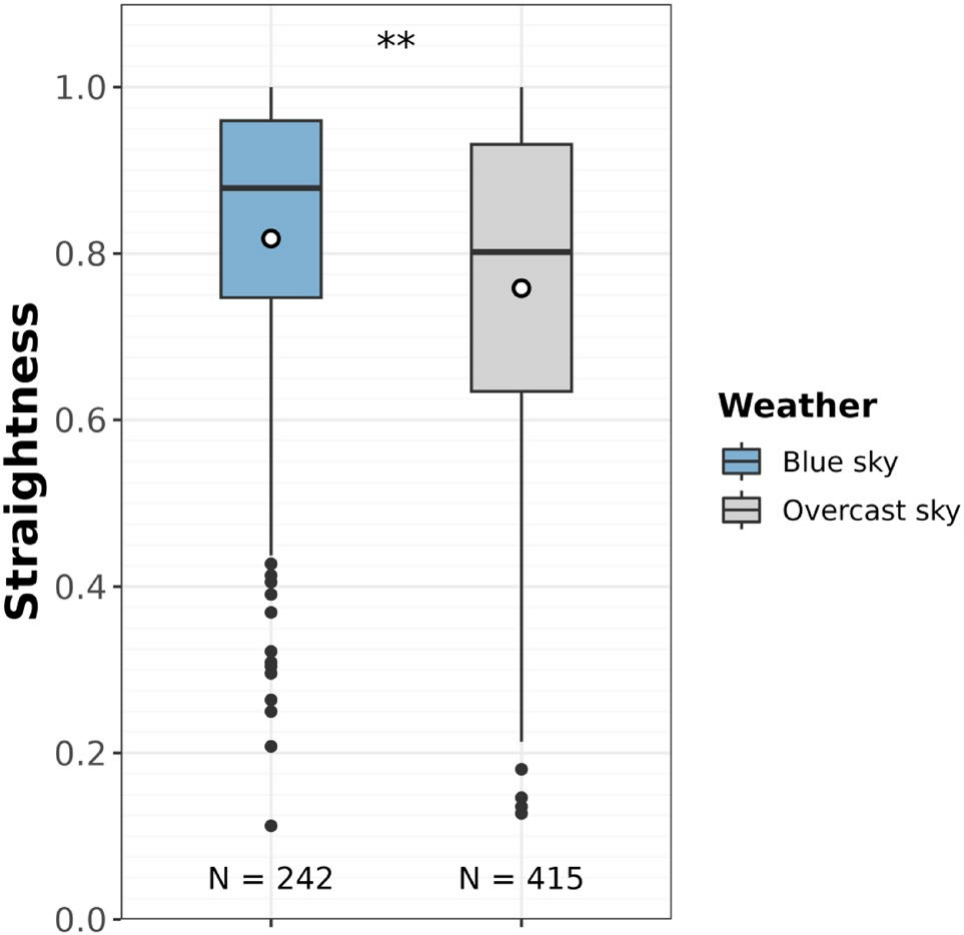
Straightness under a clear blue sky (blue) vs. overcast sky (grey)**.** The mean is represented by a white dot. The sample sizes are indicated below each boxplot. The significance refers to the linear modelling which in addition to weather conditions included canopy, understorey openness, and cosine of the sun’s elevation as test predictors, and controlled for the context (swamp day or not) and the distance travelled in the movement bouts (colour figure online)

To investigate whether the visibility of the sun may improve the navigation efficiency, we considered the 657 movement bouts toward a potential target (food or resting site; both short- and long-distance movements) located at more than 50 m (distance assumed to correspond to the gorilla visual perception range). The lighting conditions influenced how straight gorillas moved (*full* vs *null* model comparison: ${\chi }_{4}^{2}$= 26.765, *p* < 0.001). However, in the initial full model, all initial pairwise interactions (weather and canopy openness, weather and understorey openness, and weather and cosine of the sun’s elevation), were not significant (respective likelihood ratio tests: ${\chi }_{1}^{2}$= 0.281, *p* = 0.60, ${\chi }_{1}^{2}$= 0.871, *p* = 0.35, ${\chi }_{1}^{2}$= 0.825, *p* = 0.36). Weather conditions alone also influenced how straight gorillas moved (Table 1). The straightness of movements performed under an overcast vs. blue sky was lowered by 5% (from 0.81 to 0.77) when the effects of other covariates were averaged (Fig. 4, Table 1). Similarly, gorillas travelled straighter when the sun’s elevation was low (i.e., the cosine was high, Table 1), but this was independent of the weather conditions (as the interaction was non-significant, see above). Canopy and understorey openness did not influence the straightness (Table 1).

**Table 1:** Beta regression of movement straightness as a function of sun visibility conditions (N = 657**).** Non-significant interactions were removed to ease interpretability. *Est* Estimate, *SE* Standard error, *CI*_*95%*_ the 95% confidence interval, *Df* Degree of freedom, *χ*^*2*^ Chi-squared statistics. The label of the reference of categorical variables is indicated between parentheses. Continuous variables that were scaled (to a mean of 0 and a standard deviation, SD, of 1) are indicated. Their initial mean and SD values are specified as footnotes. Significant effects are highlighted in bold

Variable	Est	SE	CI _95%_	Df	*χ*^*2*^	p-value
Intercept	0.99	0.12	[0.75,1.23]	–	–	–
Weather (Overcast)	**−0.23**	**0.08**	**[−0.38,−0.07]**	**1**	**8.301**	**0.004**
Canopy (scaled)	0.02	0.05	[**−**0.09,0.13]	1	0.15	0.70
Understorey (scaled)	0.08	0.05	[**−**0.03,0.18]	1	2.15	0.14
Cosine(Sun elevation)	**0.78**	**0.20**	**[0.38,1.18]**	**1**	**15.15**	** < 0.001**
Context (Swamp)	0.20	0.15	[**−**0.09,0.49]	1	1.71	0.19
‍Distance travelled (logarithm, scaled)	**−0.23**	**0.04**	**[−0.31−0.14]**	**1**	**25.51**	** < 0.001**
Precision parameter*	3.91	0.21	[3.49,4.32]	–	–	–

Canopy (ranges from 0 to 3; prior to z-transformation): mean = 1.42, SD = 0.39

Understorey (ranges from 0 to 3; prior to z-transformation): mean = 1.52, SD = 0.43

Distance travelled, in m, prior to logarithm and z-transformation): mean = 457.84, SD = 530.71

^*^The precision relates to the estimation of the variance of the fitted beta regression

## Discussion

The navigation processes used by terrestrial mammals at the home range scale cannot be considered a simple spatial extension of those that were highlighted at a small scale in the lab (Benhamou 1996, 2010; Warren 2019), and on which an important knowledge has been accumulated in terms of behavioural and neurobiological processes (Poucet and Benhamou 1997). Our understanding of navigation at the home range scale is poorer. It has been addressed only by a few studies (e.g., Tsoar et al. 2011; Toledo et al. 2020). With this study, we aimed to offer new insights into wild western gorilla’s navigation abilities at this scale. As our results refer to only a single gorilla group, their generality may be questioned. Yet, in previous comparative studies, this group did not differ from the other study groups in terms of ranging behaviour (daily distance travelled or home range size, Robira et al. 2022), diet choice (Robira et al. 2023a, 2023b) or other behaviours (feeding and communication: Miglietta et al. 2021; dispersal and genetic patterns: Masi et al. 2021).

We showed that since the beginning of the journey, western gorillas travelled to a distant swamp in a very straight way, much straighter than the everyday movements for the days when the swamp was not visited. The swamp excursions occurred within a day and were more frequent at specific times of the year. Overall, this indicates that western gorillas memorised the swamp location and deliberately targeted it from far away (> 1500 m). All these journeys except one (see Fig. 1) started early during the day. Even though the sample size is low, this emphasises that gorillas may have planned quite quickly after the night to target the swamp, similar to female chimpanzees when foraging on widely dispersed, ephemeral, and sought-after fruit (Janmaat et al. 2014). Western gorillas seem thus able to rely on spatial memory to forage also at long distances, in addition to short-range movements as previously shown (Salmi et al. 2020; Robira, et al. 2023a; Robira et al. 2023b). As the swamp represented a unique source of mineral-rich plants (Magliocca and Gautier-Hion 2002; Metsio et al. 2014), not available elsewhere in their home range, the decision to go feeding there was likely triggered by the mineral intake need, particularly important during the high-frugivory season (Masi et al. 2015). During this period, mineral nutrient intake is indeed lower in western gorilla diet when compared to low-frugivory periods (Masi et al. 2015; Lodwick and Salmi 2019). Such a high motivation was further highlighted by the reduction of feeding and resting events on the way to the swamp. This translated into a faster travel towards the swamp from a distant starting point, because of the absence of the usual snacking and resting *en route*. Taken together, the results mean that the study gorillas efficiently navigated their home range.

Gorillas may be expected to navigate their home range using a compass and/or landmarks. For both these mechanisms, the sun may affect the way diurnal individuals may move. On one side, the sun azimuth may be used as a reference direction, but only when it is not too high (i.e., when distant from noon). We observed that around noon, western gorillas’ movements were more tortuous. However, this result was obtained irrespective of whether the sky was blue or overcast. This suggests that the apparent effect of the sun’s elevation on the straightness of directed movement was due to an (unknown) time-dependent factor rather than because the sun's elevation made it difficult to benefit from the sun azimuth, as expected if the sun was used as a compass. Certainly, hunger might play on the motivation, hence on the foraging efficiency of individuals (Noser and Byrne 2010; but see de Guinea et al. 2021 for contrasting empirical evidence). For western gorillas, noon generally represents a moment of inactivity/food satiety (Masi et al. 2009). This may result in different movement patterns associated with at least a lower speed (Salmi et al. 2020; however, note that this study used a coarser estimation of time—early/late morning/afternoon, and did not find changes in straightness along the day).

Generally, the tools used to navigate the environment vary with familiarity (Åkesson et al. 2014), which may or may not influence navigation efficiency (Presotto et al. 2019; Salmi et al. 2020). A compass is particularly useful for navigating unfamiliar areas (e.g., Guilford and Biro 2014). It is therefore not surprising that western gorillas may have not used the sun compass to navigate their home range, which corresponds to a very familiar area to them (Robira et al. 2022). The relative rarity of the movements toward the far-away swamp did not allow us to test whether the sun could be used specifically as a compass in relocation movements towards areas that are scarcely exploited by the group, thus likely also the least familiar ones. However, while these events may be infrequent over a year, visits to the swamp occur every year. Thus, some group members should have considerable experience of the whole periphery of their main range, hence being able to lead the rest of the group to the swamp from different directions. As gorilla groups are cohesive, only a few knowledgeable group members are sufficient to guide the whole group using vocalisations (Miglietta et al. 2021). This may explain the absence of preferred travelled routes towards the swamp.

The absence of compass use does not necessarily mean that a reference direction is useless for navigation; such a reference may foster efficient navigation, even in familiar environments (Benhamou 1998). At the home range scale, the visual recognition of familiar places, which should be helped by good illumination, should play a major role, particularly given primates’ visual abilities (Dominy et al. 2001; Dominy and Lucas 2001). In a familiar environment, this direction can be built up based on the configuration of surrounding landmarks, as shown by a large number of neurobiological studies performed on rats (reviewed in Poucet and Benhamou 1997). These studies highlighted not only the role of hippocampal neurons, called place cells, in the landmark-based recognition of familiar places (O'Keefe & Nadel 1978), but also of post-subiculum neurons, called head-direction cells (Taube et al. 1990a,b) in the use of a landmark-based reference direction (see model in Benhamou 1998). Thus, provided that the landmarks are sufficiently illuminated to be visually recognised, western gorillas should benefit from using a reference direction associated with the set of landmarks perceptible from the group's current position, letting a compass be unnecessary. Coherently, we showed that the lighting level affected western gorillas’ movement patterns. An overcast sky is likely to decrease visibility, contrast, and colour perception (Théry 2001), thereby landmark-based place recognition (Guilford and Graham 2014). This is probably why gorillas move straighter under a blue sky than under an overcast one. The lack of effect of the canopy and the understorey openness may, however, seem paradoxical in this regard. It can nonetheless be explained because canopy openness is usually inversely related to understorey openness as plants are competing for light access (Kitajima and Poorter 2008), so the overall ground-level brightness depends mainly on sky clarity. It now remains to elucidate how western gorillas use these landmarks to navigate. Indeed, the way landmarks are used to navigate the home range should be quite different from the landmark triangulation used to pinpoint a goal at a short distance (Benhamou 2010). At the home range scale, landmarks may be associated with the nodes of a network of routes (Poucet 1993; Benhamou 2010; Warren 2019). Many primates may indeed navigate their home range in this way (Di Fiore and Suarez 2007; Presotto et al. 2018; de Guinea et al. 2019; de Raad and Hill 2019; Abreu et al. 2021; Watkins et al. 2022).

Overall, despite being based on a single group, our study contributes to the understanding of how western gorillas navigate their home ranges. The role of sunlight in the studied gorilla group movement patterns mirrored what is observed in human populations. Humans also perform landmark-based navigation in familiar environments (Waller and Lippa 2007) and are sensitive to dark conditions, moving less accurately (Kettunen et al. 2015). Yet, despite their lower visual abilities in the dark, many so-called diurnal primates are also active at night, such as chimpanzees (Tagg et al. 2018). During this nocturnal active time, some chimpanzees have been observed navigating the forest efficiently to reach the forest edge and raid human crops (Krief et al. 2014; Lacroux et al. 2022).

Primates might thus rely on a large palette of navigational tools, favouring some over others depending on the conditions they face. In this regard, as spatial memory (Rosati 2017) and vision (Dominy and Lucas 2001) may have played together an important role in the evolutionary success of primate lineages, and shaped their movements, comparative studies of the movement patterns of different primate species under different light, weather, and moon conditions should be of considerable interest to better understand navigation processes in diurnal primates.

## Supplementary Information

Below is the link to the electronic supplementary material.

Supplementary file1 (PDF 3357 KB)

## Data Availability

Given the conservation threat that may be associated with the possession of location data for this threatened species, the request for access and use of the raw data should be made directly to the WCS in the Republic of the Congo, the owner of the data. Analytical scripts can then be provided by BR.
