# Supplementary material for: Changes in movement patterns in relation to sun conditions and spatial scales in wild western gorillas
Source: Anim Cogn. 2024 Apr 29;27(1):37. doi: 10.1007/s10071-024-01871-9 (PMC11058680; doi:10.1007/s10071-024-01871-9)
Supplement: Supplementary file 1 — Supplementary file1 (PDF 3357 KB) [file 10071_2024_1871_MOESM1_ESM.pdf]

Supplementary Material to: ‘Changes in movement patterns in relation to sun conditions and spatial scales in wild western gorillas’

Benjamin Robira[1,2,*] Simon Benhamou[1,3] Erlich Obeki Bayanga[4]
Thomas Breuer[5,6] Shelly Masi[2]

[1] Centre d’Écologie Fonctionnelle et Évolutive, Université de Montpellier & CNRS, Montpellier, France.
[2] Eco-Anthropologie, Centre National de la Recherche Scientifique/Muséum National d’Histoire Naturelle, University Paris Diderot, Sorbonne Paris Cité, Musée de l’Homme, Paris, France.
[3] Associated to Cogitamus Lab.
[4] Mondika Research Center; Nouabalé-Ndoki National Park, Wildlife Conservation Society; Congo Program, Republic of the Congo.
[5] Wildlife Conservation Society, Global Conservation Program, New-York, USA.
[6] World Wide Fund for Nature, Berlin, Germany.
[*] Correspondence: Benjamin Robira <benjamin.robira@normalesup.org>

Contents

1 Distribution of "cloudy/rainy" days along the year 3

2 Distribution of distances to the swamp at start of swamp days 3

3 Comparing observations associated with the swamp or other days 5

3.1 Timing and duration of visits to the aquatic areas 6

3.2 Longer daily travelled distances when the swamp is visited 6

3.3 Reduced feeding/resting opportunities when *en route* to the swamp 9

4 Model stability and assumptions 12

4.1 Model implementation, assumption, and stability. 12

4.2 Visual assessment of necessary assumptions 13

4.2.1 "Visit timing" models 13

4.2.2 "DPL" model 15

4.2.3 "Feeding event" model 16

4.2.4 "Resting event" model 17

4.2.5 "Sun visibility" (Straightness) model 18

4.3 Assessment of model stability 19

1 Distribution of "cloudy/rainy" days along the year

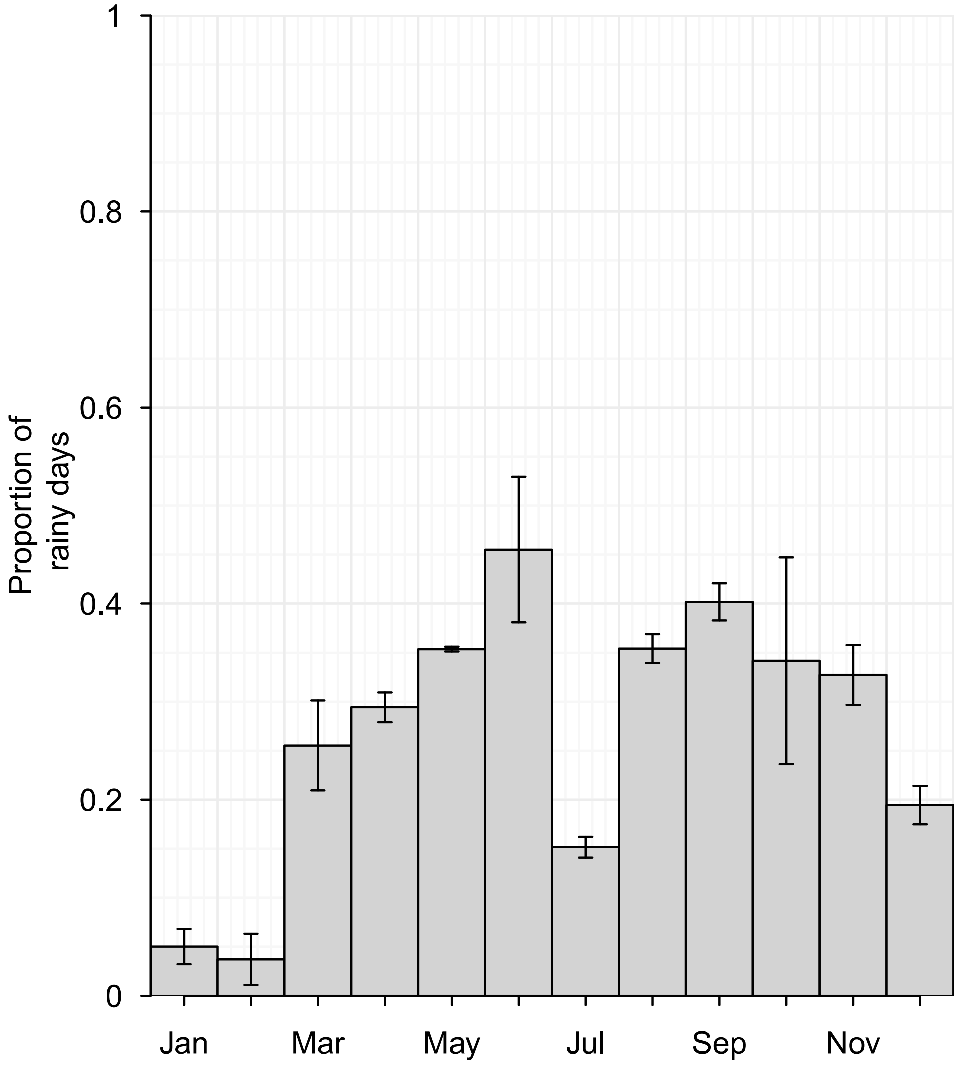

Figure 1: Distribution of rainy days along the year | The histogram displays the average proportion of rainy days occuring each month across years. The standard error is represented by the vertical error bar.

2 Distribution of distances to the swamp at start of swamp days

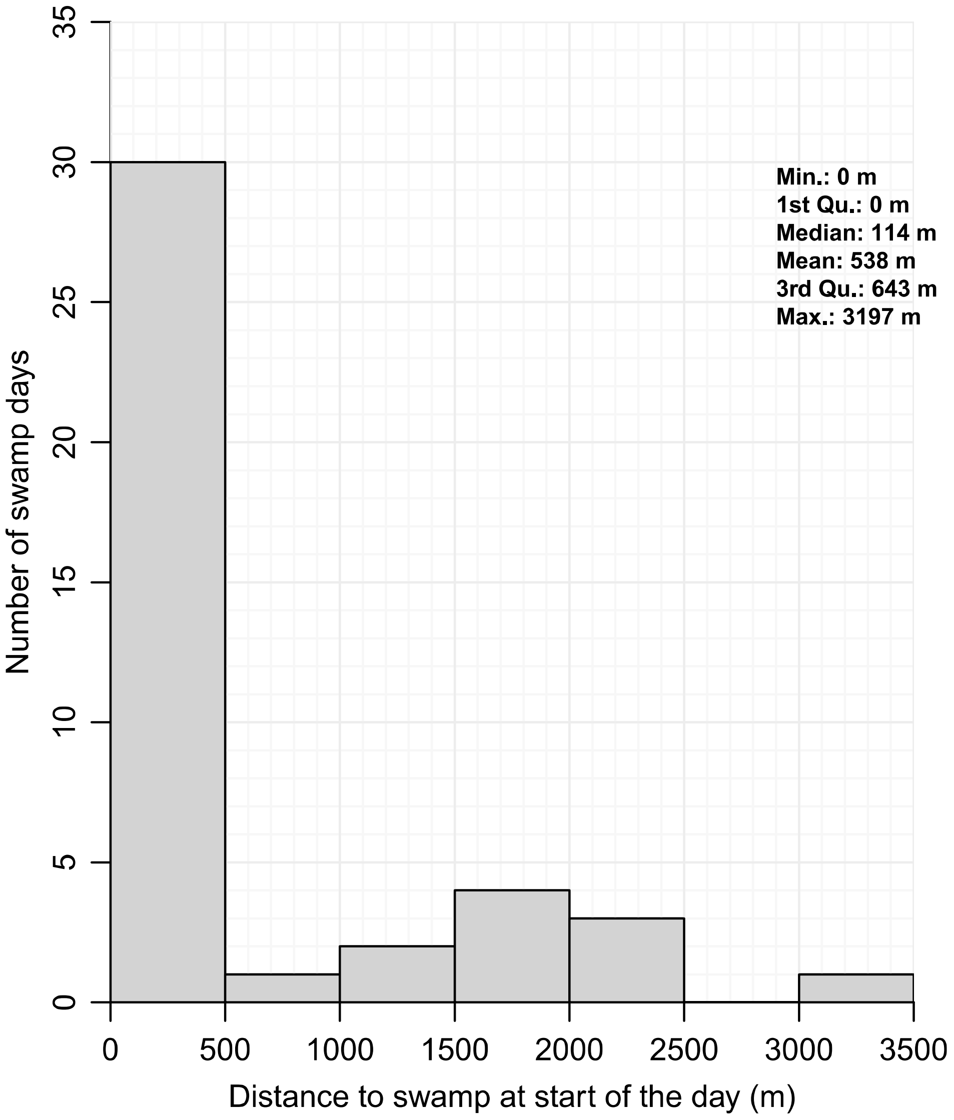

Figure 2: Distribution of distances to the swamp at the start of swamp days

3 Comparing observations associated with the swamp or other days

To emphasise the singularity of gorilla behaviour associated with the swamp, we compared observations when the swamp was visited, when a river (the other aquatic habitat found in the group home range core) was visited, or none of the two. We focused in particular on (1) gorilla diet (2) visit patterns of aquatic habitats (3) travelled distance (4) feeding/resting events.

3.1 Timing and duration of visits to the aquatic areas

We considered that a visit to one of the aquatic habitat occurred whenever the group spent more than 10 min within it, but counted it as a new visit only if the group did not visited this area within the previous 12 h [based on the rationale used by Benhamou and Riotte-Lambert, 2012]. The distribution of visits is shown in Figure 3. Considering only fully monitored years, visits to the swamp peaked in July, with an average estimation of 5.56 visits in July (Figure 3; Table 1, linear model: $\chi^2 = 6.82$, df = 1, p = 0.009). By contrast, visits to the riparian area not obviously peaked at a given time in the year (Figure 3; Table 1, linear model: $\chi^2 = 2.66$, df = 1, p = 0.103).

Average visit duration was significantly higher at the swamp than at the riparian area (median [1st,3rd quartile] : 110 [30,286] vs. 24 [12,35] min; Wilcoxon Rank Sum Test: $N_{swamp} = 41$, $N_{river} = 43$, W = 1428, $p < 0.001$). In the swamp, 17% of visits lasted several days, while this only happened once in the riparian area.

3.2 Longer daily travelled distances when the swamp is visited

To evidence that the swamps were deliberately targeted, hence reached over a singular day and not as a consequence as a drift over consecutive days followed by opportunistic targeting, we calculated the daily path length (DPL) separately for the days when gorillas visited the swamp, the riparian area or some other feeding areas. We expected that if deliberately planned, days implying visits to the swamp should thus imply longer travelled distance given the swamp is distant from gorillas core range. To do so, we compared the logarithms (to symmetrise the distribution and consider multiplicative error term) of the DPL between these three types of day using a linear model (Model 'DPL') implemented with the "lm" function of the stats sub-package of the *base* package. To take into account the differences in the duration of daily observations, we added to the model the logarithm of this duration, but we nevertheless removed days when monitoring was too low (< 6 h, unless for swamp days as given the distance from the camp to the swamp, and impossibility of travelling at night, trackers had to leave gorillas earlier those days). As gorillas tend to be reluctant to travel under heavy rain, we considered the day-weather as an additional control variable (normalised to a mean of 0 and a standard deviation of 1 in order to facilitate comparisons). A rainy day was considered when observation of rains occurred at elast once during the day. Only the 484 days involving > 6 hours of gorilla tracks were considered for analyses.

The mean DPL significantly depended on movement context, i.e. whether the gorillas visited the swamp, the riparian area, or other feeding sites ($\chi^2 = 112.14$, df = 2, $p < 0.001$, Figure 4). It was significantly larger (by 1988 m - 339% more - and 1506 m - 215% more - based on model estimation, respectively) for days when the gorillas visited the swamp than for days when they visited the riparian or other feeding sites (Table 1). The presence of rainy events during the day was associated with shorter DPL (Table 1). Overall, this suggests that gorillas made longer journeys to reach the swamp, coherently with planned movements.

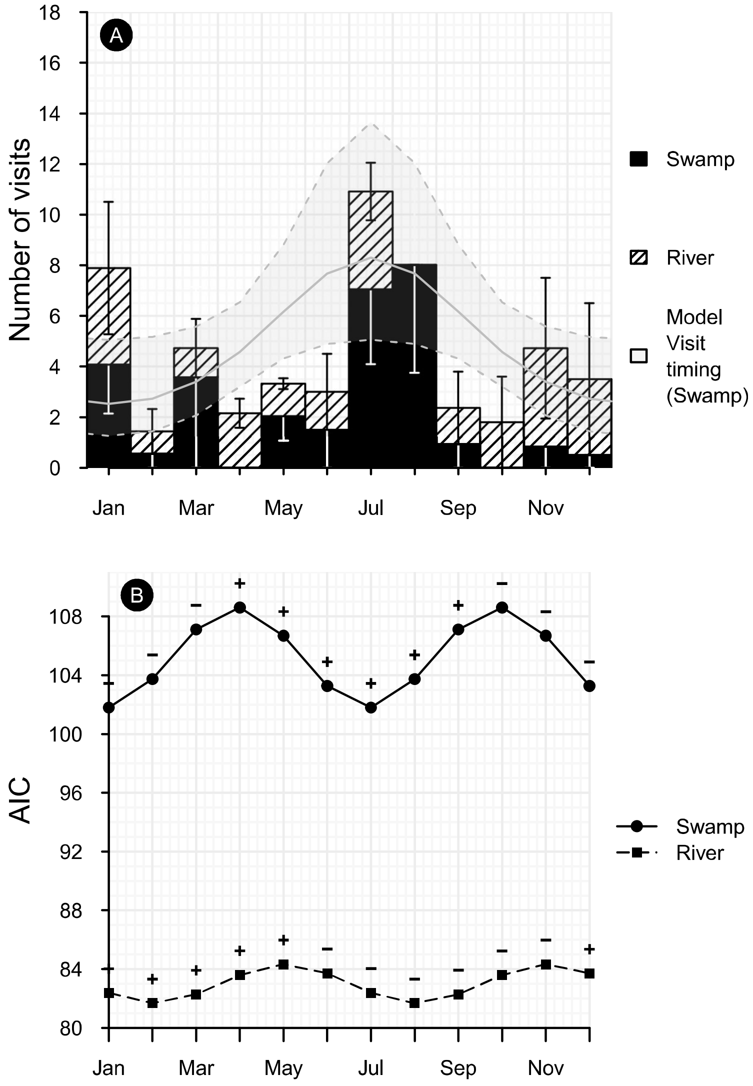

Figure 3: Timing of visitation of aquatic areas: the swamp, but not the riparian area, is seasonally targeted | (A) Average number of visits (given the chosen parameterization, only one visit per day can be counted at best) to the swamp or the riparian area. Vertical bars indicate the standard deviation. For readability, only the inferior half bar is shown for the swamp area. The estimate for the swamp model (when averaged for other covariate/offset term) is depicted by the plain line. The associated 95% confidence interval is depicted by the transparent grey background and the dashed borders. Linear models correspond to the number of visits as a function of the cosine of the month, provided the month included ten monitored days, ensuring sufficient sampling. Specifically, we computed the distribution of the ratio number of visits over number of monitoring days per month over the two years 2013-2014. We fitted a Poisson model (Visit timing model) separately for both areas (swamp and river), including a cosine (tested variable) to account for temporal oscillation such as $v_i = exp(\beta_0 + \beta_1 cos(m_i + \theta) + \beta_2 I_{2014}(y_i) + ln(d_i) + \epsilon_i)$ where y is the year and I is the indicator function being null if $y_i = 2014$. The associated Gaussian error is represented by ϵ_i. The phase θ corresponds to the month for which the maximum /minimum (depending on β_1 sign) is reached. It was set to minimise the Akaike Information Criterion [AIC, Burnham and Anderson, 2002], see (B). θ indicates that the maximum ($\beta_1 > 0$) or the minimum ($\beta_1 < 0$) of visits occurred for that month. For instance, having $\theta = 0$ would mean that the signal is the highest (or lowest, depending on β_1 sign), in January. (B) AIC of generalized linear models as a function of the month. The plotted negative and positive signs indicate the sign of the estimated slope of the cosine.

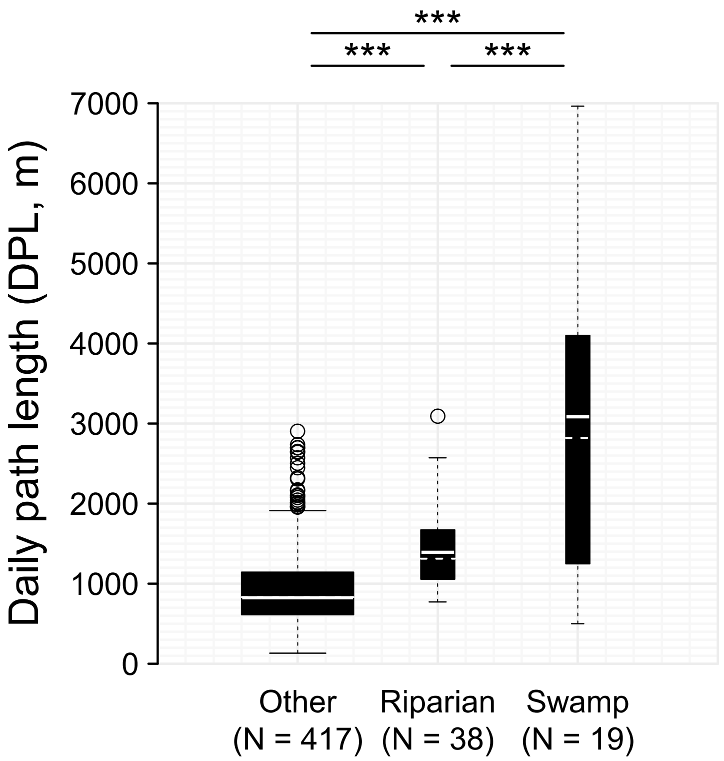

Figure 4: DPL increases on swamp days | Boxplots of the DPL for movements to the swamp, or between out-of-sight feeding sites, on days when the riparian area was visited or only non-aquatic areas were visited ('other'). The width of the box is proportional to the square root of the sample size. The median is depicted by the plain horizontal segment. The box indicates the interquartile range (IQR), and the whiskers (i.e., the vertical segment) depict the highest and lowest points within the IQR extended in both directions (upper and lower) by 1.5 times the IQR. Outlier points, which fall outside the whiskers, are represented by open circles. The linear model estimate, when the effect of other covariates is averaged, is depicted by the white dashed segment in the box.

3.3 Reduced feeding/resting opportunities when *en route* to the swamp

To further confirm that the"long" travels to the swamp were associated with a relocation strategy , we investigated the difference in feeding and resting events (> 50% of individuals , juvenile or above, resting or socializing provided three individuals were observed). We expected under a relocation strategy that gorillas engaged less in feeding and resting in order to reach the target more quickly . For this purpose , we counted (1) the number of feeding and resting events for long-distance travels towards the swamp and occurring prior to the entrance into the swamp or (2) on days when the swamp was not visited , and only for days with more than 6 hours of observations (N_{tot} = 463). We fitted a Poisson model ("Feeding event"/"Resting event" models) with a log-link function using the "glm" function, with the number of feeding or resting/social events as the response variable and the context (swamp day, riparian day, or non-aquatic days) as a categorical predictor . We considered the logarithm of the traveled distance as a control predictor, to account for the fact that the longer the travel, the more feeding events would be expected, all else being equal. We scaled this variable to a mean of 0 and a standard deviation of 1, to ease interpretability [Schielzeth , 2010]. We initially wanted to include it as an offset term (i.e., no estimated slope), but this induced deviation from necessary model assumptions. In the "Resting model ", some deviations occurred (i.e., statistical significance of associate control tests) but seemed minor (Figure 10).

The results of the models are shown in Figure 5 and detailed in Table 1. We observed a decrease in the number of feeding events (2.07 and 1.41 less - 69% and 60% less - for an average travel of around 1 km, based on model estimation) when gorillas travelled towards the swamp than days they visited the riparian area (reference is the swamp, est. = 1.17, CI95% = [0.72,1.67]) or only non-aquatic areas (reference is the swamp, est. = 0.92, CI95% = [0.49,1.42]), respectively. We observed a decrease in the number of resting events (2.48 and 3.92 less - 62% and 72% - for an average travel of around 1 km based on model estimation) when gorillas travelled towards the swamp than days they visited the riparian area (reference is the swamp, est. = 0.97, CI95% = [0.38,1.68]) or only non-aquatic areas (reference is the swamp, est. = 1.28, CI95% = [0.71,1.98]), respectively. This suggests that the larger speed when moving towards the swamp is a by-product of the lower feeding rate and resting/socializing rate. In other words, gorillas switched from a foraging/exploring strategy to a travelling strategy when moving to the swamp.

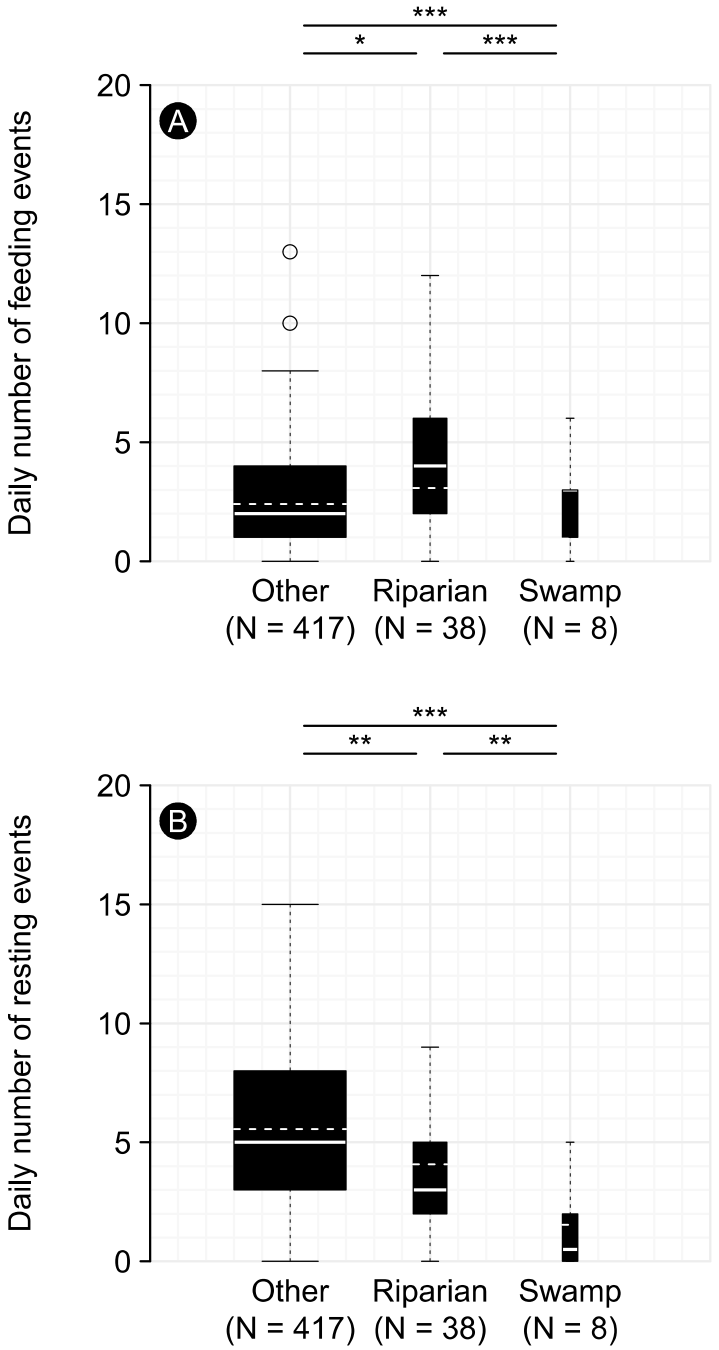

Figure 5: Gorillas spend less time feeding and resting when *en route* to the swamp | Boxplots of the number of feeding/resting events per day for movements to the swamp, or between out-of-sight feeding sites, on days when the riparian area was visited or only non-aquatic areas were visited ('other'). The width of the box is proportional to the square root of the sample size. The median is depicted by the plain horizontal segment. The box indicates the interquartile range (IQR), and the whiskers (i.e., the vertical segment) depict the highest and lowest points within the IQR extended in both directions (upper and lower) by 1.5 times the IQR. Outlier points, which fall outside the whiskers, are represented by open circles. The linear model estimate, when the effect of other covariates is averaged, is depicted by the white dashed segment in the box.

Table 1: Summary of the generalized linear regressions | Est. = Estimate, SE = Standard error, CI95% = the 95% confidence interval, Df = Degree of freedom, Chi²/F = Chi-squared/Fisher statistics. Not available values or with no meaningfulness are not displayed (i.e., '-' symbol). The level for categorical variables is indicated between parentheses. Continuous variables that were scaled are indicated. Their initial mean and sd are depicted in footnotes.

Variable	Est.	SE	CI95%	Df	Chi²/F	p-value
**Model Visit Swamp: (N = 24)**						
Intercept	-2.66	0.17	[-3.02,-2.34]	-	-	-
cos(month + best phase)	**-0.6**	**0.23**	**[-1.07,-0.15]**	**1**	**6.82**	**0.009**
Year (2014)	-0.25	0.17	[-0.58,0.07]	1	2.28	0.131
**Model Visit River: (N = 24)**						
Intercept	-3.18	0.3	[-3.82,-2.64]	-	-	-
cos(month + best phase)	0.4	0.25	[-0.08,0.9]	1	2.66	0.103
Year (2014)	0.69	0.36	[-5.06e-03,1.42]	1	3.78	0.052
**Model DPL (in m): (N = 474)**						
Intercept	6.72	0.02	[6.68,6.77]	-	-	-
Vegetation (river)	**0.46**	**0.08**	**[0.29,0.62]**	**2**	**62.59**	**<0.001**
Vegetation (swamp)	**1.22**	**0.12**	**[0.98,1.46]**	**2**	**62.59**	**<0.001**
Duration monitoring (in hour, scaled)	**0.11**	**0.02**	**[0.06,0.16]**	**1**	**20.78**	**<0.001**
Weather (scaled)	**0.07**	**0.02**	**[0.03,0.12]**	**1**	**10.15**	**0.002**
**Model Feeding: (N = 463)**						
Intercept	0.88	0.03	[0.81,0.94]	-	-	-
Vegetation (river)	**0.24**	**0.09**	**[0.07,0.41]**	**2**	**31.93**	**<0.001**
Vegetation (swamp)	**-0.92**	**0.24**	**[-1.42,-0.49]**	**2**	**31.93**	**<0.001**
Distance travelled (logarithm, scaled)	**0.42**	**0.03**	**[0.36,0.49]**	**1**	**175.01**	**<0.001**
**Model Resting: (N = 463)**						
Intercept	1.72	0.02	[1.67,1.76]	-	-	-
Vegetation (river)	**-0.31**	**0.09**	**[-0.49,-0.14]**	**2**	**35.21**	**<0.001**
Vegetation (swamp)	**-1.28**	**0.32**	**[-1.98,-0.71]**	**2**	**35.21**	**<0.001**
Distance travelled (logarithm, scaled)	**-0.1**	**0.02**	**[-0.14,-0.05]**	**1**	**20.15**	**<0.001**

[a] Model DPL; Duration (in hour, prior to z-transformation): mean = 8.3, sd = 1

[b] Model DPL; Weather (prior to z-transformation): mean = 0.74, sd = 0.44

[c] Model Feeding/Resting; Distance travelled (in meter, prior to log/z-transformation): mean = 1025, sd = 589

4 Model stability and assumptions

4.1 Model implementation, assumption, and stability.

We assessed the influence of the test predictors as a whole using a likelihood-ratio test ("lrtest" function of the *lmtest R* package [Zeileis and Hothorn, 2002]), comparing the *full* model with a *null* model only including a constant term and the control variable(s), and if multiple variables were tested, we compared the fit on the *full* model with that of a reduced model with a likelihood ratio-test, discarding one variable at a time (i.e., "drop1" function, or manually done for beta-regression). Finally, for categorical predictors, we computed pairwise comparisons by running the model several times changing the reference level. In this case, p-values were corrected with a Bonferroni correction ("p.adjust" function, method set to "bonferroni").

In brief, for all models we checked basic assumptions [distribution of residuals, Q-Q plot, homoscedasticity and overdispersion if adequate; Zeileis and Hothorn [2002]] using the *DHARMa R* package [Hartig, 2022]. Knowing that the statistical assessment are very sensitive, visual inspection further confirmed that no major issues were found (see Visual assessment of necessary assumptions). We verified model stability based on several statistical indicators [Cook's distance, leverage, DfBetas, Quinn and Keough, 2003, Zuur et al., 2009]. They pointed out the existence of no major influential case either (see Assessment of model stability). Possible correlations between variables were not problematic [maximum Variance Inflation Factor, VIF, Field, 2005, close to 1, "vif" function from the *car R* package Fox and Weisberg [2019]].

4.2 Visual assessment of necessary assumptions

4.2.1 "Visit timing" models

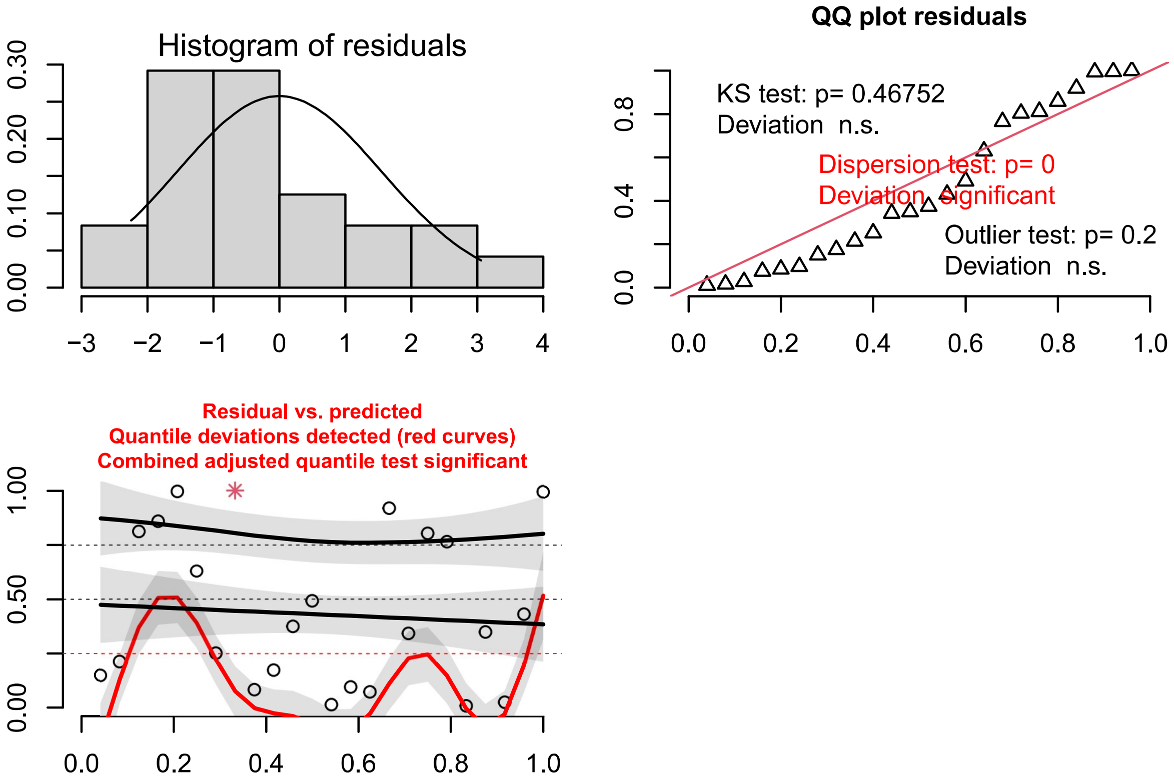

Figure 6: Model assumptions check: 'Visit timing' models (when AIC minimised; swamp model) | Depicted are the histogram of residuals, the Q-Q plot (adapted for the modeled distribution; with test for deviation of the distribution (Kolmogorov-Smirnov, KS, outliers and overdispersion) and the scatter plot of the fitted values vs. the residuals. Plots were based on the *DHARMa* package [Hartig, 2022].

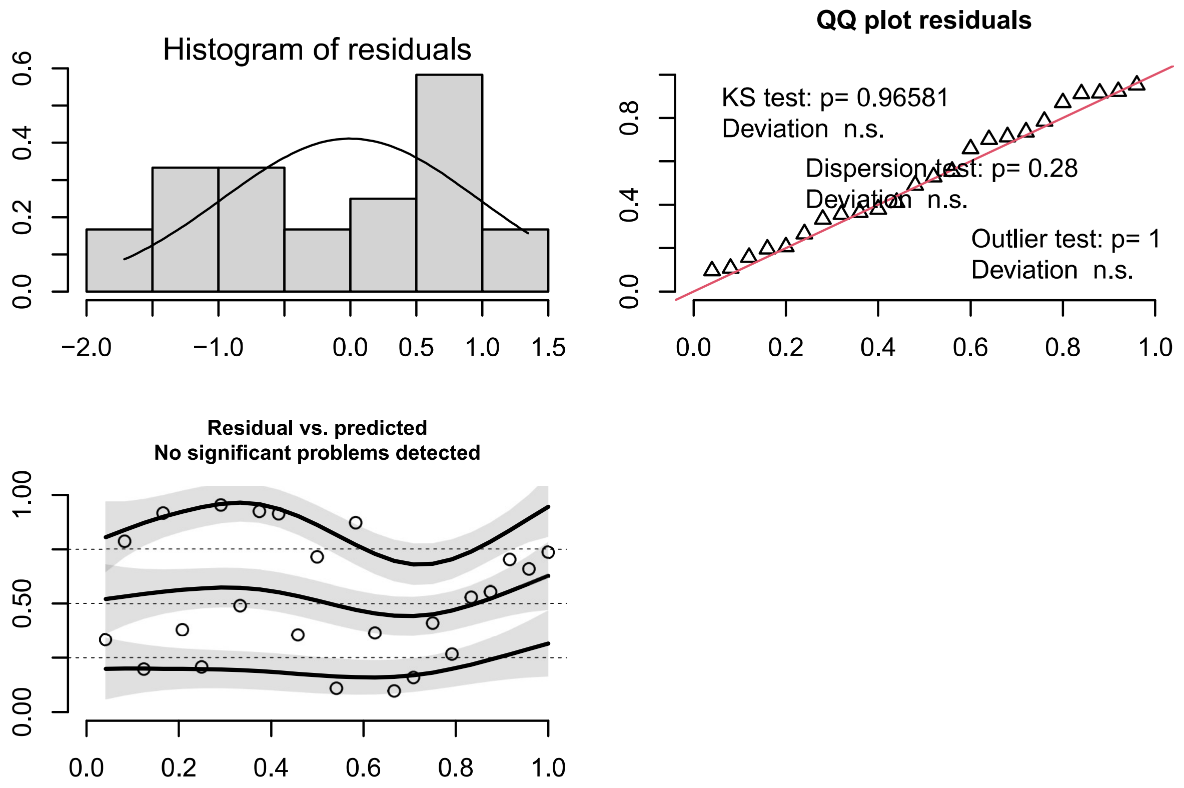

Figure 7: Model assumptions check: 'Visit timing' models (when AIC minimised; riparian model) | Depicted are the histogram of residuals, the Q-Q plot (adapted for the modeled distribution; with test for deviation of the distribution (Kolmogorov-Smirnov, KS, outliers and overdispersion) and the scatter plot of the fitted values vs. the residuals. Plots were based on the *DHARMa* package [Hartig, 2022].

4.2.2 “DPL” model

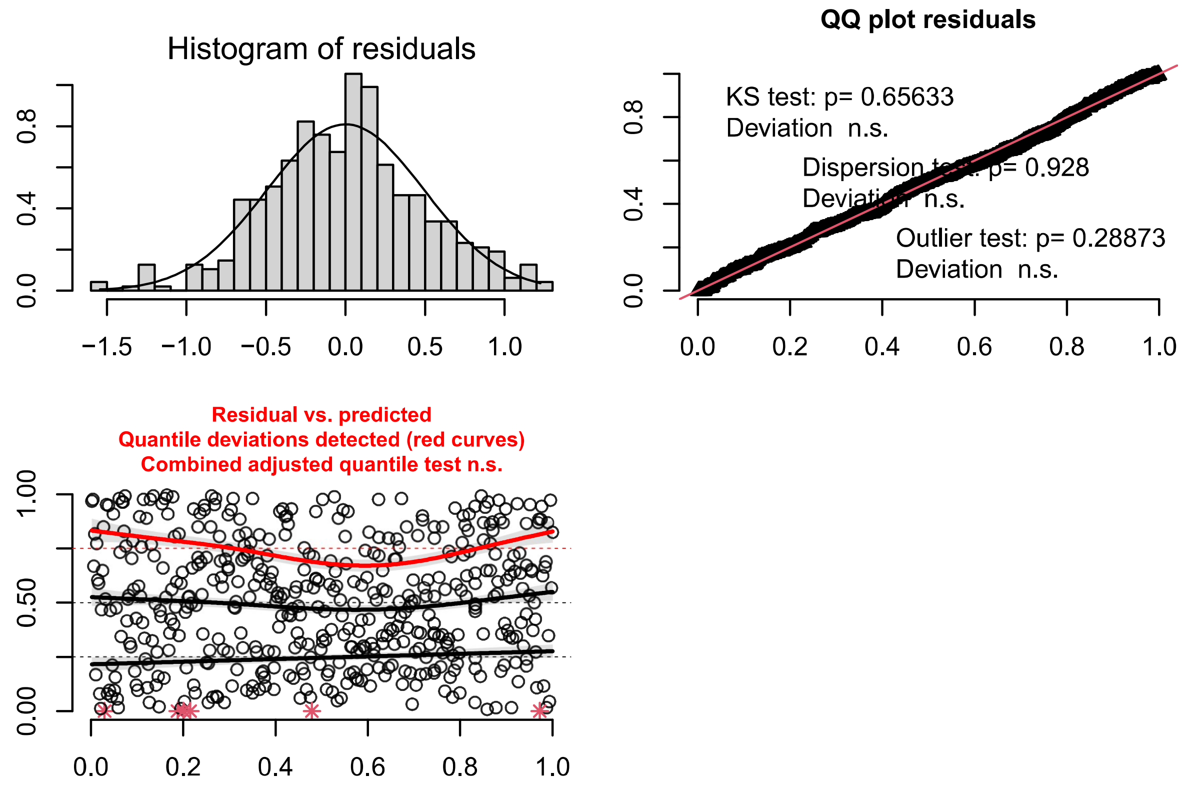

Figure 8: Model assumptions check: ‘DPL’ model | Depicted are the histogram of residuals, the Q-Q plot (adapted for the modeled distribution; with test for deviation of the distribution (Kolmogorov-Smirnov, KS, outliers and overdispersion) and the scatter plot of the fitted values vs. the residuals. Plots were based on the *DHARMa* package [Hartig, 2022].

4.2.3 “Feeding event” model

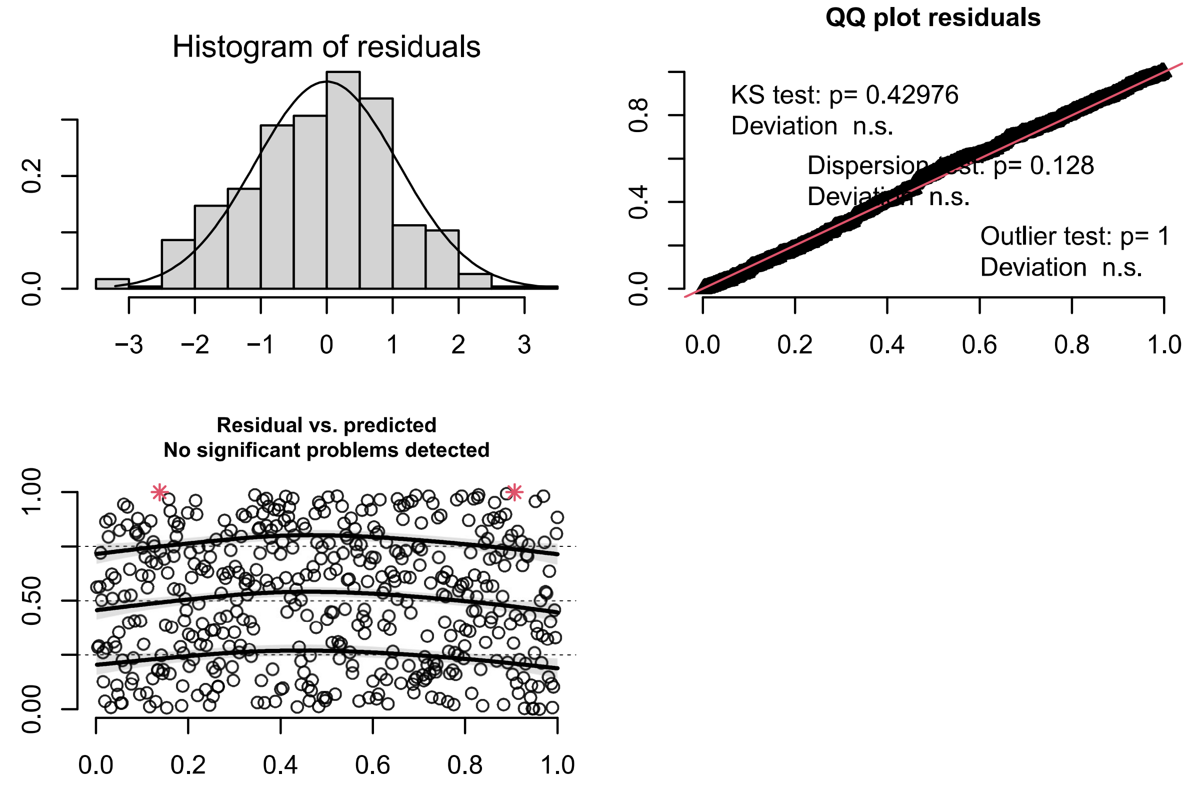

Figure 9: Model assumptions check: ‘Feeding event’ model | Depicted are the histogram of residuals, the Q-Q plot (adapted for the modeled distribution; with test for deviation of the distribution (Kolmogorov-Smirnov, KS, outliers and overdispersion) and the scatter plot of the fitted values vs. the residuals. Plots were based on the *DHARMa* package [Hartig, 2022].

4.2.4 "Resting event" model

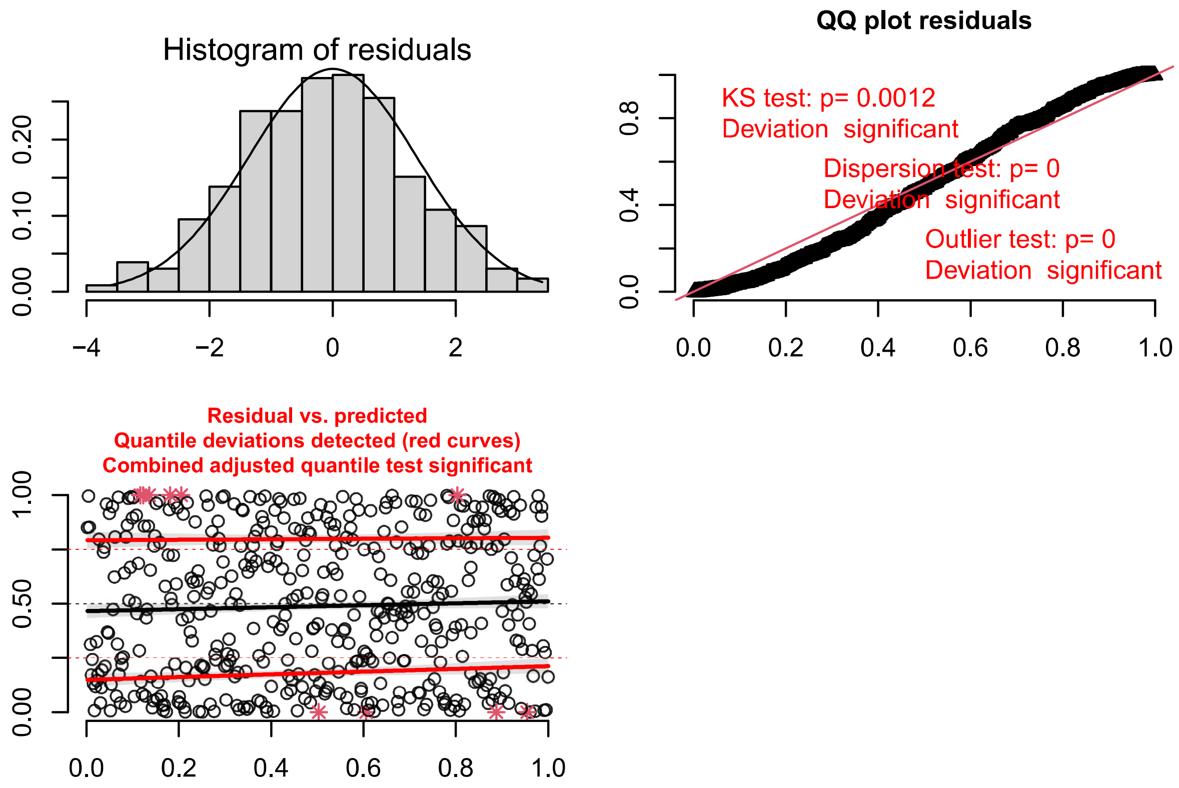

Figure 10: Model assumptions check: 'Resting event' model | Depicted are the histogram of residuals, the Q-Q plot (adapted for the modeled distribution; with test for deviation of the distribution (Kolmogorov-Smirnov, KS, outliers and overdispersion) and the scatter plot of the fitted values vs. the residuals. Plots were based on the *DHARMa* package [Hartig, 2022].

4.2.5 "Sun visibility" (Straightness) model

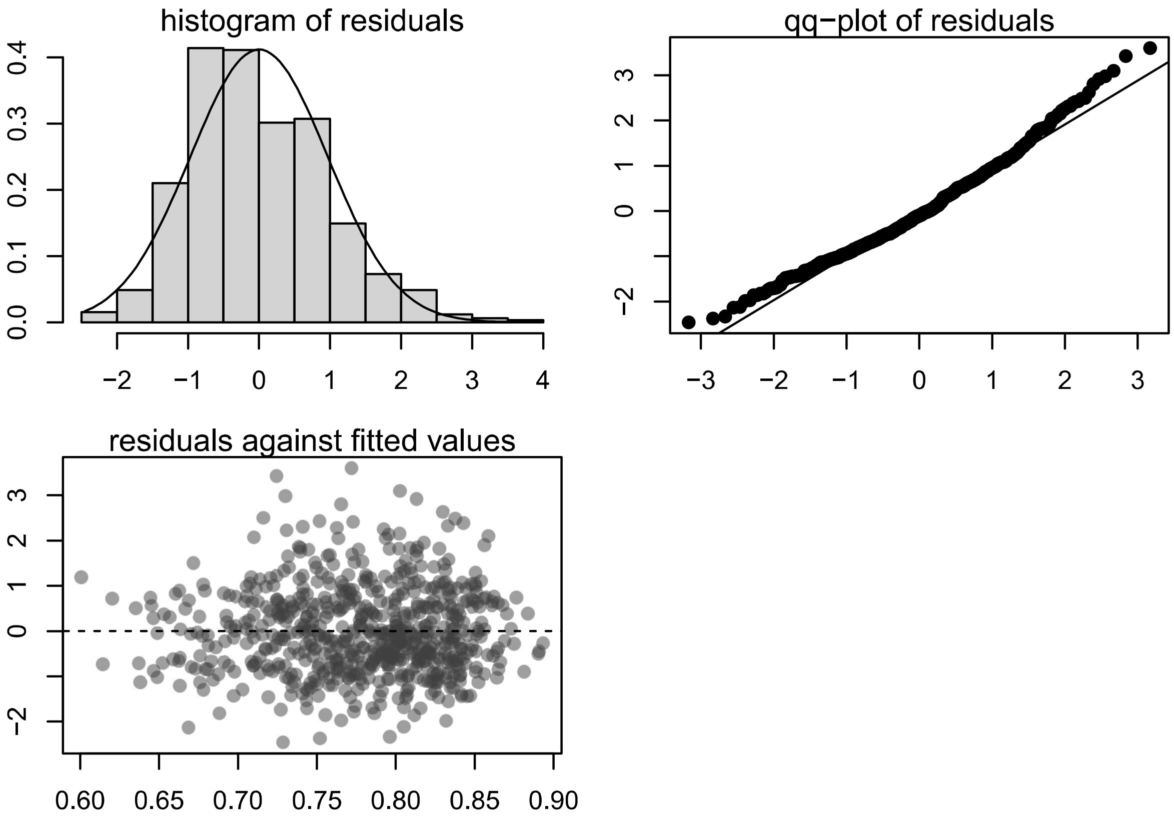

Figure 11: Model assumptions check: 'Model assumptions check: 'Sun azimuth (Straightness)' model | Depicted are the histogram of residuals, the Q-Q plot and the scatter plot of the fitted values vs. the residuals. Plots were based on handmade function.

4.3 Assessment of model stability

Table 2: Summary of the DFBetas for all linear models but the beta regressions | For visit models, the reference level is year 2013. For remaining models, the reference level is when neither the swamp or the riparian forest was visited.

	Est.	Min	Max
'Visit Timing Swamp' Model:			
Intercept	-2.42	-2.57	-2.21
Cosine of month	-0.6	-0.82	-0.38
Year 2014	-0.48	-0.77	-0.27
'Visit Timing River' Model:			
Intercept	-3.18	-3.41	-3.04
Cosine of month	0.4	0.25	0.57
Year 2014	0.69	0.57	0.94
'DPL' Model:			
Intercept	6.72	6.72	6.73
River	0.46	0.44	0.48
Swamp	1.22	1.15	1.28
Monitoring duration (scaled)	0.11	0.1	0.12
Weather (scaled)	0.07	0.07	0.08
'Feeding Event' Model:			
Intercept	0.88	0.87	0.88
River	0.24	0.2	0.27
Swamp	-0.92	-1.11	-0.77
Distance travelled (scaled)	0.42	0.41	0.43
'Resting Event' Model:			
Intercept	1.72	1.71	1.72
River	-0.31	-0.35	-0.28
Swamp	-1.28	-1.51	-0.97
Distance travelled (scaled)	-0.1	-0.1	-0.08

Table 3: Statistical indicators of stability for linear models

	Cook's distance	Leverage (max. hat value)	Max. DFFit	Max. VIF
Model Visit Timing Swamp	1.22	0.22	1	1.01
Model Visit Timing River	0.35	0.25	0.71	1.01
Model DPL	0.1	0.1	0.7	2
Model Feeding	0.17	0.17	0.75	2
Model Sun elevation (Straightness)	0.04	0.03	-	2.14

References

Simon Benhamou and Louise Riotte-Lambert. Beyond the Utilization Distribution: Identifying home range areas that are intensively exploited or repeatedly visited. *Ecological Modelling*, 227:112–116, 2012. ISSN 03043800. doi: 10.1016/j.ecolmodel.2011.12.015.

Kenneth P. Burnham and David R. Anderson. *Model selection and multimodel inference: A practical Information-theoretic approach*. Springer, New York, second edition, 2002.

Andy Field. *Discovering statistics using SPSS*. Sage publications, 2005.

John Fox and Sanford Weisberg. *An R Companion to Applied Regression*. Sage, Thousand Oaks CA, third edition, 2019. URL https://socialsciences.mcmaster.ca/jfox/Books/Companion/.

Florian Hartig. *DHARMa: Residual Diagnostics for Hierarchical (Multi-Level / Mixed) Regression Models*, 2022. URL https://CRAN.R-project.org/package=DHARMa. R package version 0.4.6.

Gerry P. Quinn and Michael J. Keough. *Experimental design and data analysis for biologists*, volume 28. Cambridge University Press, 2003.

Holger Schielzeth. Simple means to improve the interpretability of regression coefficients. *Methods in Ecology and Evolution*, 1(2):103–113, 2010. doi: 10.1111/j.2041-210x.2010.00012.x.

Achim Zeileis and Torsten Hothorn. Diagnostic checking in regression relationships. *R News*, 2(3):7–10, 2002. URL https://CRAN.R-project.org/doc/Rnews/.

Alain Zuur, Elena N Ieno, Neil Walker, Anatoly A Saveliev, and Graham M Smith. *Mixed effects models and extensions in ecology with R*. Springer Science & Business Media, 2009.
